# Novel nomograms based on microvascular invasion grade for early-stage hepatocellular carcinoma after curative hepatectomy

**DOI:** 10.1038/s41598-024-54260-0

**Published:** 2024-02-12

**Authors:** Hengkai Chen, Honghao Ye, Linfang Ye, Fangzhou Lin, Yingjun Shi, Aoxue Zhong, Guoxian Guan, Jinfu Zhuang

**Affiliations:** 1 Department of Colorectal Surgery, The First Affiliated Hospital of Fujian Medical University, 20th, Chazhong Road, Fuzhou, 350005 China; 2 Department of Colorectal Surgery, National Regional Medical Center, Binhai Campus of the First Affiliated Hospital, Fujian Medical University, Fuzhou, 350212 China; 3 Fuzhou University, Fuzhou, 350108 China; 4 Mengchao Hepatobiliary Hospital of Fujian Medical University, Fuzhou, 350025 China; 5 Zhongshan Hospital Xiamen University, Xiamen, 361004 China

**Keywords:** Nomogram, Microvascular invasion grade, Early-stage HCC, Early recurrence, Cancer, Cancer models

## Abstract

Microvascular invasion (MVI) is a critical risk factor for postoperative recurrence of hepatocellular carcinoma (HCC). This study aimed to firstly develop and validate nomograms based on MVI grade for predicting recurrence, especially early recurrence, and overall survival in patients with early-stage HCC after curative resection. We retrospectively reviewed the data of patients with early-stage HCC who underwent curative hepatectomy in the First Affiliated Hospital of Fujian Medical University (FHFU) and Mengchao Hepatobiliary Hospital of Fujian Medical University (MHH). Kaplan–Meier curves and Cox proportional hazards regression models were used to analyse disease-free survival (DFS) and overall survival (OS). Nomogram models were constructed on the datasets from the 70% samples of and FHFU, which were validated using bootstrap resampling with 30% samples as internal validation and data of patients from MHH as external validation. A total of 703 patients with early-stage HCC were included to create a nomogram for predicting recurrence or metastasis (DFS nomogram) and a nomogram for predicting survival (OS nomogram). The concordance indexes and calibration curves in the training and validation cohorts showed optimal agreement between the predicted and observed DFS and OS rates. The predictive accuracy was significantly better than that of the classic HCC staging systems.

## Introduction

Hepatocellular carcinoma (HCC) is among the most frequent causes of cancer-related deaths worldwide^1^. Despite remarkable improvements in comprehensive HCC treatment, radical surgical resection and liver transplantation are considered the only curative treatments for patients with HCC classified as early-stage (stages 0 and A) according to the Barcelona Clinic Liver Cancer (BCLC) staging system. However, postoperative recurrence and metastasis rates of patients with early HCC vary from 50 to 70%^2^, resulting in poor overall survival (OS). Early recurrence after liver resection for HCC is the leading cause of death during the first 2 years^3^. Therefore, developing a model for predicting postoperative recurrence, especially early recurrence, for patients with early-stage HCC to guide risk stratification and treatment is urgently needed.

Microvascular invasion (MVI), a mass of cancer cells in the vascular cavity with adhesion to endothelial cells, and only visible under a microscope^4^, has been reported by previous studies to be an indicator of early invasive manifestation of HCC. It is a crucial independent predictive factor for early recurrence and poor OS among patients with HCC who underwent hepatectomy or received liver transplantation. Most patients with BCLC early-stage HCC with early recurrence are pathologically verified as MVI positive ^5–7^. Moreover, a previous study found that more invading tumor cells and multiple-invaded microvessels might be related to poor survival and recurrence rates^4^. These findings suggest that the BCLC staging system should reappraise HCC based on the presence or grade of MVI to distinguish the biological behavior of early-stage HCC.

MVI is graded according to the number of cancer cells and the distance of MVI to the tumor according to the Standard for Diagnosis and Treatment of Primary Liver Cancer^8^. Although predictive models for postoperative early recurrence in patients with HCC have been established, a predictive model for patients with early BCLC stage HCC patients according to the MVI grade has not been reported.

Therefore, we retrospectively investigated the clinical and histopathological characteristics of patients with early HCC after curative hepatectomy from two centers to establish a prognostic nomogram based on MVI grade to predict early recurrence and OS.

## Methods

### Patients and study design

The database was retrospectively derived from patients with HCC who underwent hepatectomy at the First Affiliated Hospital of Fujian Medical University (FHFU) and the Mengchao Hepatobiliary Hospital of Fujian Medical University (MMH) from March 2015 to March 2020.

The inclusion criteria for patients with HCC patients in this study were: (1) early-stage HCC (BCLC stage 0 or A) diagnosis that was confirmed by postoperative pathology; (2) Child–Pugh A or B liver function before surgery; (3) R0 surgical resection of the tumor with curative intent; (4) all patients who survived for at least 30 days after surgery; (5) no preoperative anticancer treatments that could introduce any bias; and (6) clinicopathological data and follow-up information were available. Patients with the following criteria were excluded: (1) recurrent HCC, (2) combined hepatocellular cholangiocarcinoma, (3) previous history of malignancy, and (4) age < 18 years. R0 surgical resection was defined as complete tumor resection with histopathologically tumor-free resection margins.

Nomogram models were constructed on the datasets from the FHFU, which were also validated using bootstrap resampling as internal validation, and the dataset from the MMH was used for external validation. This study was approved by the Ethics Review Committee of the First Affiliated Hospital of Fujian Medical University and the Ethics Review Committee of Mengchao Hepatobiliary Hospital of Fujian Medical University. Written informed consent was obtained from all subjects before the operation. All procedures were performed in accordance with the Declaration of Helsinki.

### Clinical variables

**Figure 1:**
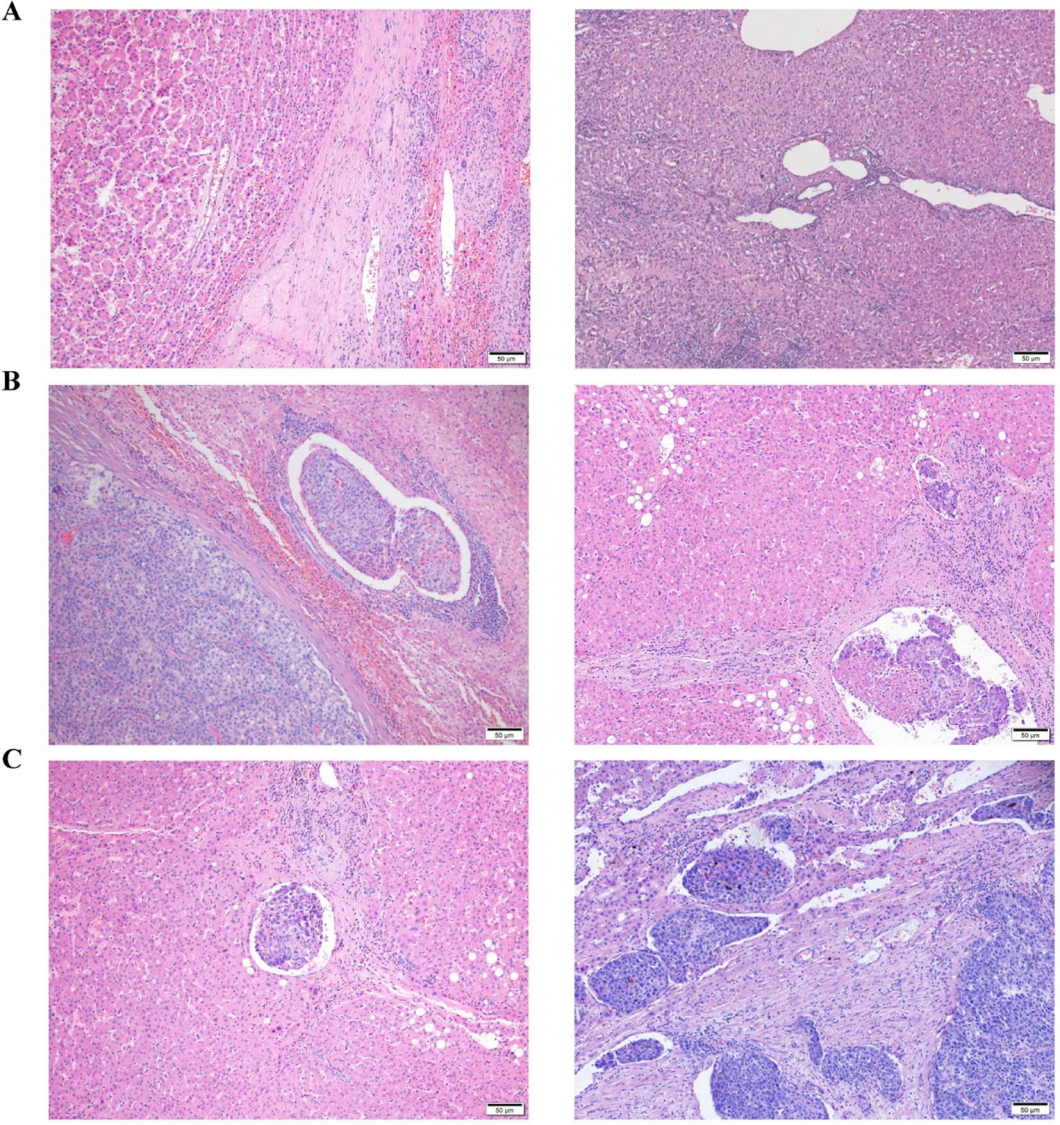
The grade of MVI Standard for Diagnosis and Treatment of Primary Liver Cancer. (**a**) M0: no MVI; (**b**) M1: the number of MVI is < 5 and at a distance of ≤ 1 cm from the tumor; (**c**) M2: the number of MVI is > 5 or at a distance of > 1 cm from the tumor.

Demographic, laboratory, and HCC pathological data were collected. The laboratory tests included various tests for routine blood parameters, full sets of tests for blood clotting, full sets of tests for blood biochemistry, and hepatitis virus markers. Imaging data included, but were not limited to, the number of tumors, presence of satellite nodules, the diameter of the largest nodule, tumor capsule, and cirrhosis based on preoperative contrast-enhanced computed tomography (CT) or magnetic resonance imaging (MRI). The diagnosis and grade of MVI were confirmed according to the Standard for Diagnosis and Treatment of Primary Liver Cancer^4^ by 2 independent pathologists. In case of any doubt, the final decision was determined after MDT discussion. Briefly, the grade of MVI is defined as follows: M0: no MVI; M1: the number of MVI is < 5 and at a distance of ≤ 1 cm from the tumor; M2: the number of MVI is > 5 or at a distance of > 1 cm from the tumor (Fig. 1)^4^.

### Follow-up

During the follow-up, serum alpha-fetoprotein (AFP) levels were measured, and ultrasonography, CT, or MRI of the chest and abdomen was done once every 2 months for the first 2 years after surgery. For patients who were free of cancer recurrence 2 years after surgery, a 6-month interval surveillance was performed. Disease-free survival (DFS) was defined as the duration from the first surgery to the first recurrence, metastasis, or death. OS was defined as the duration from the first surgery to death or the last follow-up.

### Statistical analysis

Continuous variables are expressed as mean ± standard deviation. Chi-squared or Fisher’s exact tests were used to assess differences in categorical variables. The Wilcoxon rank-sum test was used to compare continuous variables between groups. In this study, the cut-off values of continuous variables were established using the X-tile software version 3.6.1 (Yale University School of Medicine, New Haven, Connecticut, United States) for widely clinical application. For DFS and OS curves during follow-up, Kaplan–Meier curves, log-rank Mantel-Cox test, and Cox proportional hazards regression analyses were used. Nomograms were generated using the rms package in R software version 3.5.2 (R Foundation for Statistical Computing, Vienna, Austria)^9^. The predictive accuracy and discriminative ability of the nomogram were assessed using the concordance index (C-index) and calibration curves. The larger the C-index, the more accurate the prognostic prediction is. A value of *P* < 0.05 was considered significant. All covariates met the Cox proportional hazard assumption, as determined by the Schoenfeld residuals. Variance inflator factor was used to assess multicollinearity in all estimated models; there was no indication of multicollinearity.

### Ethics approval and consent to participate

This study was approved by the Ethics Review Committee of the First Affiliated Hospital of Fujian Medical University and the Ethics Review Committee of Mengchao Hepatobiliary Hospital of Fujian Medical University. Written informed consent was obtained from all subjects before the operation. All procedures were performed in accordance with the Declaration of Helsinki.

### Consent for publication

Written informed consent was obtained from every patient with HCC to perform tumor resection for analysis and publication.

## Results

### Characteristics of patients in study and validation cohorts

**Figure 2:**
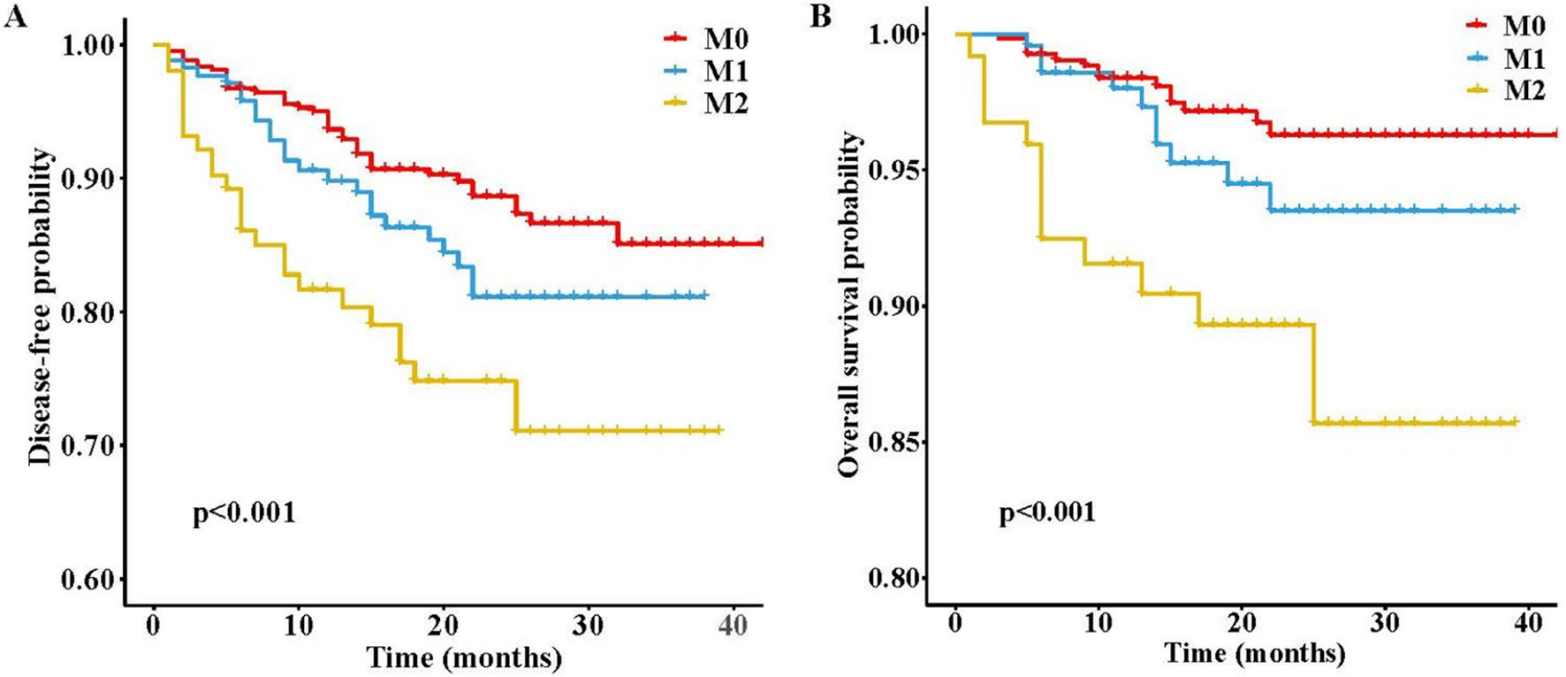
Kaplan–Meier estimates of the prognosis of patients with early-stage HCC according to MVI grade. (**a**) The MVI grade satisfactorily determined the disease-free survival (DFS) in the whole cohort; (**b**) The MVI grade satisfactorily determined the overall survival (OS) in the whole cohort.

Overall, 703 patients (490 from FHFU used as training cohort, and 213 from MMH used as external validation cohort) with BCLC early-stage HCC were included. The baseline characteristics of the two cohorts are shown in Supplementary Table 1. The average age of the entire cohort was 53.7 ± 10.9 years, with a male to female ratio of 4.72:1 (580/130). The average follow-up time for all patients was 18.8 ± 10.2 months. The 8-month, 1-, 2-, and 3-year recurrence or metastasis rates were 5.8%, 8.1%, 11.8%, and 12.7%, respectively. The 8-month, 1-, 2-, and 3-year survival rates were 1.7%, 2.4%, 3.9%, and 4.2%, respectively. Among them, M1 was observed in 173 cases (24.6%), while M2 was observed in 102 cases (14.5%). Between-group differences in sex, age, operative time, follow-up time, ASA scores, laboratory, and HCC pathological data were not significant (Supplementary Table 1). The association of MVI grade relative to OS or DFS is shown in Fig. 2. The Kaplan–Meier curves of OS and DFS showed that the M2 group had significantly poorer outcomes than the M1 and M0 groups (both *P* < 0.001).

The univariate analysis results for DFS and OS in the study cohort are shown in Table 1. Multivariate analysis revealed that eight factors including neutrophils, alkaline phosphatase (ALP), urea, low-density lipoprotein (LDL), apolipoprotein A1 (Apo-A1), thrombin time (TT), tumor size, and MVI grade were independent prognostic factors for DFS, while six factors including TT, MVI grade, mean corpuscular haemoglobin (MCH), monocyte, prealbumin (PAB) and α-fucosidase (AFU) were prognostic factors for OS (Table 1). Therefore, these variables were included in the subsequent analysis to establish predictive models.

**Table 1:** Univariate and multivariate of clinical parameters associated with DFS and OS in early-stage HCC patients after R0 resection.

Clinical parameter	DFS		OS	
	HR (95% CI)	*p* value	HR (95% CI)	*p* value
Univariate analysis				
Age, year	0.25 (0.08–0.77)	0.015	0.98 (0.95–1)	0.091
Sex, male/female	0.81 (0.45–1.46)	0.998	0.88 (0.41–1.9)	0.741
BCLC staging system, (0/A)	3.04 (0.75–12.33)	0.122	2.74 (0.68–11.29)	0.995
WBC, ≤ 3.7/ > 3.7 × 10^9^/L	2.41 (0.98–6.02)	0.055	1.21 (1.06–1.5.2)	0.013
PLT, ≤ 160/ > 160 × 10^9^/L	1.76 (1.12–2.77)	0.018	1.00 (1.00–1.00)	0.027
Hb, ≤ 125/ > 125 × 10^9^/L	2.05 (0.81–5.06)	0.133	0.99 (0.97–1.12)	0.312
Hematocrit, ≤ 42.2/ > 42.2%	1.56 (0.67–2.32)	0.073	0.98 (0.91–1.12)	0.692
MCV, ≤ 87/ > 87fL	0.66 (0.42–1.03)	0.061	0.95 (0.90–1.01)	0.055
MCH, ≤ 31.5/ > 31.5 pg	0.45 (0.28–0.72)	< 0.001	0.85 (0.76–0.96)	0.007
Lymphocyte, ≤ 1.5/ > 1.5 × 10^9^/L	0.93 (0.60–1.45)	0.732	0.86 (0.52–1.55)	0.585
Neutrophil, ≤ 3.3/ > 3.3 × 10^9^/L	2.02 (1.01–3.80)	0.045	1.43 (1.14–1.72)	0.002
Monocyte, ≤ 0.5/ > 0.5 × 10^9^/L	1.54 (0.89–2.60)	0.126	7.51 (1.63–15.86)	0.012
LR, ≤ 41.6/ > 41.6%	0.56 (0.33–0.96)	0.036	0.95 (0.92–0.99)	0.011
NR, ≤ 47.8/ > 47.8%	1.60 (0.97–2.52)	0.068	1.05 (1.00–1.10)	0.033
RDW, ≤ 13.6/ > 13.6%	1.81 (1.12–3.01)	0.016	1.12 (0.89–1.56)	0.292
RBC, ≤ 4.5/ > 4.5 × 10^9^/L	1.35 (0.86–2.63)	0.152	1.22 (0.66–2.24)	0.533
AFP, < 400/ ≥ 400 μg/L	2.01 (1.20–3.26)	0.005	1.00 (1.00–1.00)	< 0.001
ALT, < 61/ ≥ 61 U/L	13.12 (1.85–5.40)	< 0.001	1.00 (0.99–1.00)	1.000
HBV DNA				
< 50/ ≥ 50 × 10^9^ IU/mL	1.74 (1.12–2.96)	0.016	1.00 (1.00–1.00)	0.971
Albumin, < 50/ ≥ 50 g/L	0.47 (0.23–0.93)	0.031	0.89 (0.82–0.97)	0.008
Scr, < 76/ ≥ 76 μmol/L	0.62 (0.34–1.12)	0.123	0.98 (0.96–1.02)	0.240
γ-GGT, < 34/ ≥ 34U/L	2.10 (1.12–3.95)	0.021	1.00 (1.00–1.00)	0.541
ALP, < 76/ ≥ 76& < 117 ≥ 117U/L	4.75 (2.61–8.56)	< 0.001	1.00 (1.00–1.00)	0.182
TBil, < 16.4/ ≥ 16.4 μmol/L	1.44 (0.88–2.12)	0.169	0.97 (0.93–1.24)	0.313
DBil, < 5.6/ ≥ 5.6 μmol/L	1.52 (0.97–2.30)	0.065	0.95 (0.83–1.10)	0.415
IBil, < 6.6/ ≥ 6.6 μmol/L	1.55 (0.83–2.70)	0.178	0.94 (0.86–1.00)	0.182
TBA, < 7.2/ ≥ 7.2 μmol/L	2.33 (1.20–4.32)	0.011	0.95 (0.89–1.01)	0.061
TP, < 77 / ≥ 77 g/L	0.26 (0.07–1.01)	0.049	1.00 (0.95–1.10)	0.706
ALB, < 37/ ≥ 37 g/L	1.40 (0.23–0.93)	0.031	0.89 (0.82–0.97)	0.008
GLB, < 28.2/ ≥ 28.2 g/L	1.42 (0.88–2.10)	0.168	1.00 (0.96–1.06)	0.727
ALB/GLB < 2.2/ ≥ 2.2	0.39 (0.19–0.82)	0.013	1.73 (0.26–1.30)	0.186
PAB, ≤ 200/ > 200 mg/L	0.32 (0.14–0.73)	0.007	0.99 (0.99–1.00)	0.0014
AFU, < 38/ ≥ 38 g/L	4.12 (1.62–10.04)	0.003	1.02 (1.00–1.03)	0.032
ADA, < 7/ ≥ 7U/L	1.92 (1.25–3.04)	0.008	1.12 (0.99–1.32)	0.063
LDH, < 212/ ≥ 212U/L	2.02 (1.12–3.76)	0.033	1.00 (1.00–1.00)	< 0.001
Urea, < 4/ ≥ 4& < 6.9/ ≥ 6.9 mmol/L	0.22 (0.08–0.59)	0.003	0.95 (0.76–1.22)	0.623
Uric acid, < 379/ ≥ 379 μmol/L	1.64 (0.99–2.62)	0.053	1.01 (0.99–1.03)	0.192
GLU, < 4.9/ ≥ 4.9 mmol/L	0.60 (0.38–0.94)	0.025	0.97 (0.78–1.22)	0.821
TCHO, < 3.4/ ≥ 3.4 mmol/L	1.95 (0.74–4.78)	0.184	1.02 (0.75–1.40)	0.990
TG, < 1.1/ ≥ 1.1 mmol/L	0.60 (0.37–0.98)	0.043	0.51 (0.24–1.18)	0.081
HDL, < 1/ ≥ 1 mmol/L	0.70 (0.43–1.12)	0.156	1.25 (0.48–3.05)	0.702
LDL, < 3/ ≥ 3 mmol/L	2.08 (1.22–3.16)	0.005	0.98 (0.65–1.52)	0.905
Apo-A1, < 83/ ≥ 83 g/L	0.45 (0.22–0.91)	0.028	1.00 (0.99–1.01)	0.760
Apo-B, < 113/ ≥ 113 g/L	2.01 (1.14–3.66)	0.027	1.00 (0.99–1.01)	0.708
Calcium, < 2.5/ ≥ 2.5 mmol/L	1.75 (0.24–12.4)	0.590	2.01 (0.09–4.01)	0.660
Phosphorus, < 1.1/ ≥ 1.1 mmol/L	1.72 (1.03–3.01)	0.043	10.01 (1.82–20.14)	0.008
Magnesium, < 0.8/ ≥ 0.8 mmol/L	0.70 (0.38–1.25)	0.212	2.10 (0.02–4.24)	0.751
Kalium, < 4.5/ ≥ 4.5 mmol/L	1.84 (1.01–3.23)	0.044	3.72 (1.65–8.62)	0.003
Natrium, < 141/ ≥ 141 mmol/L	0.57 (0.36–0.89)	0.014	0.99 (0.87–1.15)	0.860
Chlorine, < 102/ ≥ 102 mmol/L	0.46 (0.29–0.73)	< 0.001	0.93 (0.84–1.02)	0.151
TT, < 20/ ≥ 20 s	0.46 (0.22–0.95)	0.035	0.75 (0.59–0.96)	0.025
FIB, < 2.8/ ≥ 2.8 g/L	2.10 (1.34–3.37)	0.002	1.81 (1.40–2.34)	< 0.001
APTT, < 25.7/ ≥ 25.7 s	0.59 (0.37–0.92)	0.020	1.01 (0.93–1.15)	0.960
PT, < 11.3/ ≥ 11.3 s	1.52 (0.94–2.57)	0.092	1.00 (0.76–1.41)	0.933
Tumor size, < 5/ ≥ 5& < 10/ ≥ 10centimiter	3.82 (2.12–6.84)	< 0.001	1.22 (1.14–1.35)	< 0.001
Tumor number, single/multiple	0.62 (0.20–12.0)	0.415	1.55 (0.55–4.36)	0.408
Satellite nodules, yes/no	1.72 (1.13–2.61)	0.022	1.20 (0.69–2.1)	0.525
MVI, M0/M1/M2	2.60 (1.51–4.45)	< 0.001	2.20 (1.61–3.05)	< 0.001
Tumor capsule, yes/no	0.87 (0.67–1.15)	0.308	0.62 (0.20–2.01)	0.415
Cirrhosis, yes/no	0.77 (0.46–1.32)	0.313	0.54 (0.25–1.15)	0.107
Multivariate analysis				
Neutrophil	0.34 (0.19–0.60)	< 0.001		
ALP	4.41 (2.05–9.62)	< 0.001	–	–
Urea	0.46 (0.26–0.80)	0.007	–	–
LDL	2.15 (1.34–3.67)	0.003	–	–
Apo-A1	0.32 (0.16–0.66)	0.002	–	–
TT	0.34 (0.16–0.70)	0.003	0.92 (0.87–0.97)	0.003
Tumor size	2.20 (1.29–3.82)	0.008	-	-
MVI grade	2.31 (1.25–4.16)	0.009	3.71 (1.75–7.85)	< 0.001
MCH	–	–	0.67 (0.54–0.83)	< 0.001
Monocyte	–	–	4.67 (2.37–9.68)	< 0.001
PAB	–	–	0.56 (0.38–0.84)	0.005
AFU	–	–	0.71 (0.61–0.82)	< 0.001

*BCLC staging system* Barcelona Clinic Liver Cancer staging system, *WBC* white blood cell, *PLT* platelet, *Hb* hemoglobin, *MCV* mean corpuscular volume, *MCH* mean corpuscular hemoglobin, *LR* lymphocyte ratio, *NR* neutrophil ratio, *MR* monocyte ratio, *RDW* red blood cell distribution width, *MPC* mean platelet volume, *RBC* red blood cell, *AFP* α-fetoprotein, *ALT* alanine aminotransferase, *HBV DNA level* hepatitis B virus deoxyribonucleic acid level, *Scr* Serum creatinine, *γ-GTT* γ-glutamyl transpeptidase, *ALP* alkaline phosphatase, *TBil* total bilirubin, *DBil* direct bilirubin, *IBil* indirect bilirubin, *TBA* total bile acid, *TP* total protein, *ALB* albumin, *GLB* globumin, *PAB* prealbumin, *AFU* α-fucosidase, *ADA* adenosine deaminase, *LDH* lactate dehydrogenase, *GLU* glucose, *TCHO* total cholesterol, *TG* triglyceride, *HDL* high-density lipoprotein, *LDL* low-density lipoprotein, *Apo-A1* apolipoprotein A1, *Apo-B* apolipoprotein B, *TT* thrombin time, *FIB* fibrinogen, *APTT* activated partial thromboplastin time, *PT* prothrombin time, *MVI* microvascular invasion.

### Establishment of nomogram model for postoperative early-relapse/OS and evaluation of its discriminability and calibration

**Figure 3:**
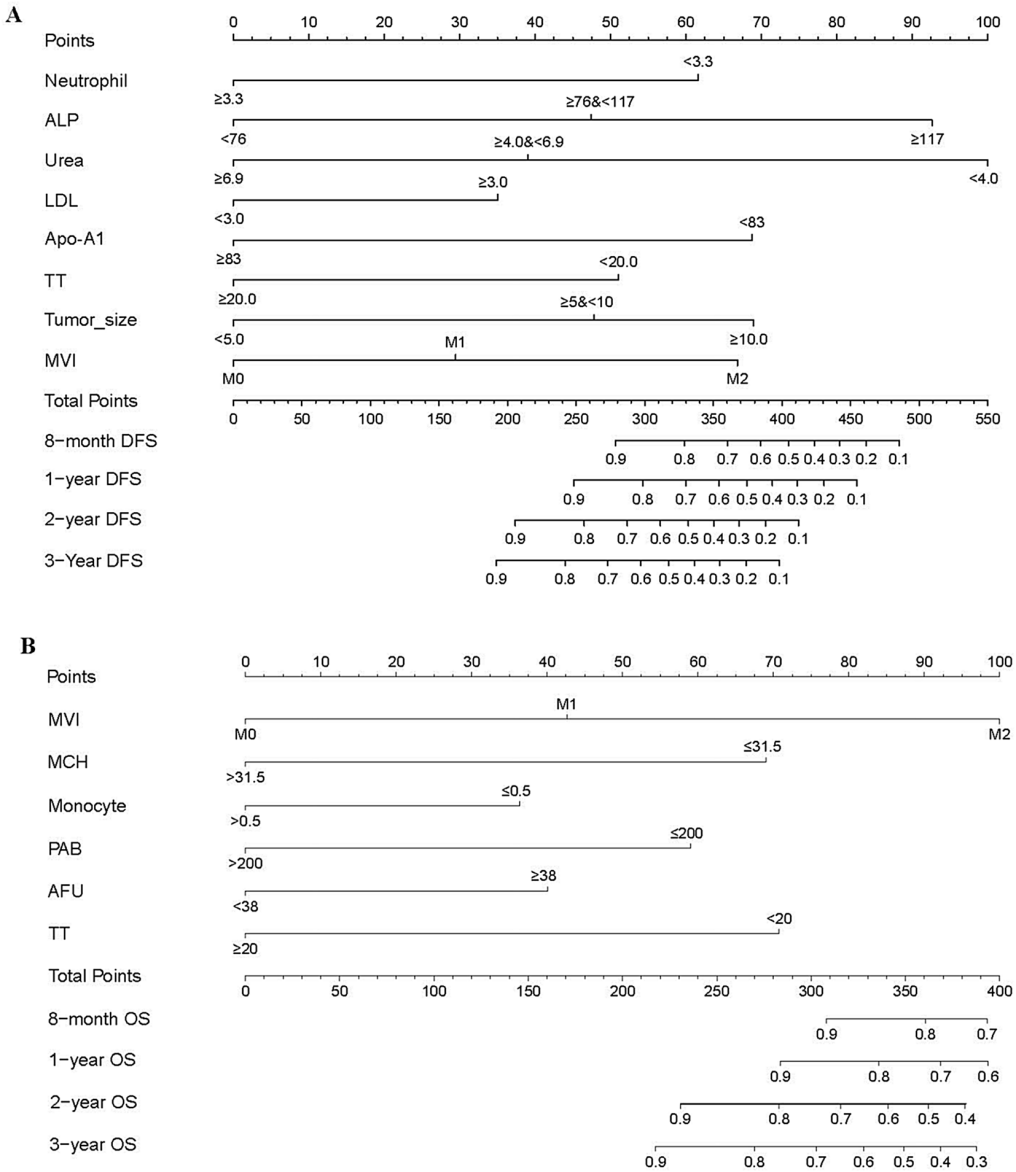
Nomograms for predicting disease-free survival (DFS) and overall survival (OS) in patients with early-stage HCC after curative hepatectomy. (**a**) DFS; (**b**) OS. MCH, mean corpuscular hemoglobin; ALP, alkaline phophatase; PAB, prealbumin; AFU, α-fucosidase; Apo-A1, apolipoprotein A1; TT, thrombin time; MVI, microvascular invasion.

**Figure 4:**
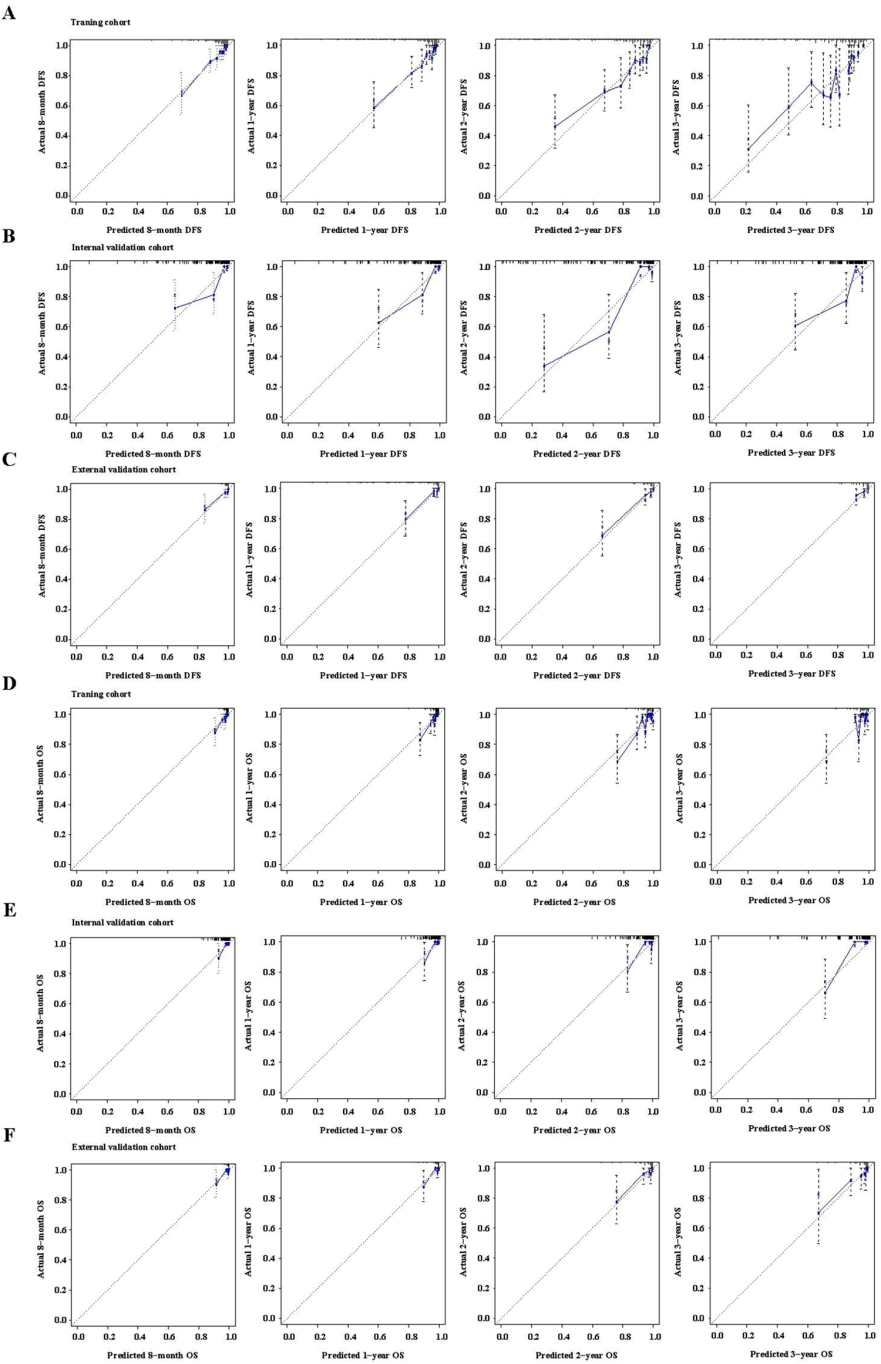
Calibration curves for predicting disease-free survival (DFS) and overall survival (OS) using the nomograms. (**a**) 8-month, 1, 2, and 3-year DFS in the training cohort; (**b**) 8-month, 1, 2, and 3-year DFS in the internal validation cohort; (**c**) 8-month, 1, 2, and 3-year DFS in the external validation cohort; (**d**) 8-month, 1, 2, and 3-year OS in the training cohort; (**e**) 8-month, 1, 2, and 3-year OS in the internal validation cohort; (**f**) 8-month, 1, 2, and 3-year OS in the external validation cohort.

Based on the independent prognostic factors, nomograms for DFS and OS in the study cohort were established (Fig. 3). The results are shown in Supplementary Table 2. The C-index of the nomogram for DFS was 0.775 (95% confidence interval [CI] 0.720–0.830). The C-index for OS was 0.812 (95% CI 0.732–0.892). The validation showed excellent consistency between the observed and predicted 8-month, 1-, 2-and 3-year DFS, and 8-month, 1-, 2- and 3-year OS (Fig. 3); with a C-index of 0.865 (95% CI 0.806–0.924) for DFS and a C-index of 0.839 for OS (95% CI 0.675–1.00) in the internal validation cohort, and with a C-index of 0.857 (95% CI 0.763–0.951) for DFS and a C-index of 0.842 (95% CI 0.708–0.970) for OS in the external validation cohort (Supplementary Table 2). Calibration curves of internal verification and external verification with slopes closed to 1 and all p-value greater than 0.05 in the Hosmer and Lemeshow test, showed good consistency between the observed and predicted events (Fig. 4). Taken together, the nomogram models were able to accurately predict postoperative relapse and OS in patients with BCLC early-stage HCC.

### Comparison of predictive accuracy between the nomogram models and the classical staging systems

The predictive value of the constructed model, in terms of clinical practicability, was compared with that of the 8th edition American Joint Committee on Cancer (AJCC) staging system, the BCLC staging system, the Japan Integrated Staging Score (JIS) and the Hong Kong Liver Cancer prognostic classification scheme (HKLC). The results are shown in Supplementary Table 2. In the training cohort, the C-index of the nomogram for DFS and OS was 0.775 and 0.812, respectively, which was significantly higher than the AJCC (DFS: 0.591; OS: 0.588), BCLC (DFS: 0.601; OS: 0.599), JIS (DFS: 0.589; OS: 0.592), and HKLC (DFS: 0.595; OS: 0.612) staging systems. Similarly, in the validation cohort, the C-index of the nomogram for DFS (internal cohort: 0.865; external cohort: 0.857) and OS (internal cohort: 0.839; external cohort: 0.842), was also significantly higher than the AJCC (internal cohort: 0.622, external cohort: 0.586 for DFS; and internal cohort: 0.615, external cohort: 0.578 for OS), BCLC (internal cohort: 0.602, external cohort: 0.574 for DFS; and internal cohort: 0.608, external cohort: 0.571 for OS), JIS (internal cohort: 0.606, external cohort: 0.581 for DFS; and internal cohort: 0.599, external cohort: 0.574 for OS), HKLC (internal cohort: 0.625, external cohort: 0.558 for DFS; and internal cohort: 0.619, external cohort: 0.541 for OS) staging systems. Overall, the nomogram models exhibited superior predictive accuracy to that of these authoritative staging systems for DFS and OS.

## Discussion

Although patients with BCLC early-stage HCC typically have a more favorable prognosis compared to those with late-stage HCC characterized by macrovascular invasion and multiple intrahepatic metastases, a significant proportion of patients still experience recurrence and metastasis. The presence of macrovascular invasion is universally recognized as a highly influential factor in predicting poor prognosis among patients with early-stage HCC^4,8,9^. Moreover, recent research has demonstrated a strong correlation between the grade of macrovascular invasion and postoperative recurrence, particularly early recurrence^8,10–13^. Neither of the two most commonly used pathological staging systems for hepatocellular carcinoma (HCC) incorporates the presence of MVI as a criterion. Currently, there is a lack of reported predictive models utilizing the MVI grading system to identify patients with early-stage HCC who are at a high risk of recurrence or have a poor prognosis. The development of such a model would be advantageous in order to establish a system for early and continuous monitoring or prompt postoperative adjuvant therapy for HCC patients. Consequently, nomograms were developed utilizing the MVI grading system to predict recurrence and overall survival in early-stage HCC patients who underwent curative surgery. Subsequent validation demonstrated a favorable concordance between the nomogram predictions and observed outcomes in terms of predictive probability. Furthermore, our nomograms exhibited superior predictive efficacy compared to the conventional BCLC and AJCC staging systems.

The prognosis of patients with HCC is mainly affected by: (1) patient factors, such as immune function, nutritional state, liver function, and status of hepatitis virus infection; (2) tumor factors, such as tumor diameter, MVI classification, and satellite nodules; and (3) factors of treatment, in particularly adjuvant treatment after surgery. In our study, nine of the twelve risk factors associated with recurrence or OS were patient factors, including neutrophil, monocyte, ALP, PAB, MCH, Urea, LDL, Apo-A1, and TT levels, while three factors were tumor-related factors including tumor size, MVI classification, and AFU. These results indicate that the prognosis of HCC is a multifactorial and complex process.

The histopathological types and grades of MVI serve as indicators of the histopathological transformations that transpire when a cancer embolus within a vessel develops into a satellite lesion or a metastatic site. Consequently, the histopathological type of MVI can be employed as a morphological marker for assessing the biology and advancement of HCC^4,14,15^. The detectability rate of MVI in patients with early-stage HCC ranges from 12.4% to 33.1%, and the prognostic significance of MVI in this patient population following curative surgery is still a matter of debate^16–18^. In our study, we found that MVI was an independent risk factor associated with DFS and OS (Fig. 2, *P* < 0.001), with a detection rate of 39.1% (275/703). Recently, studies indicated that the tumor microenvironment in MVI-positive HCC patients was more immunosuppressive than that in MVI-negative HCC patients. This environment could promote tumor progression by activating signaling pathways that enhance tumor cell proliferation, migration, and angiogenesis including HIF-1 pathway, Wnt pathway, MAPK pathway, and Ras pathway^19–21^. Additionally, it recruits inhibitory immune cells and upregulates immune checkpoints to mediate immune escape in tumors^22–24^. Ultimately, these mechanisms jointly contribute to recurrence and metastasis in MVI-positive HCC patients after curative hepatectomy. The relationship between tumor size and patient prognosis is widely acknowledged, particularly in cases of HCC. Tumor enlargement has been consistently associated with a poor prognosis in HCC patients, leading to the establishment of cut-off values in various guidelines to predict prognosis. This is due to the non-linear nature of the relationship between tumor size and poor prognosis. For the purpose of this study, the cut-off values of 5 and 10 cm were utilized. Notably, our study revealed that tumors with a diameter exceeding 10 cm were identified as a significant risk factor for recurrence. Interestingly, despite AFP being widely recognized as a conventional clinical marker for diagnosing and prognosticating patients with HCC, our study found that it did not independently correlate with prognosis in early-stage HCC following curative hepatectomy. This observation may be attributed to the limited sensitivity of AFP in predicting the prognosis of early-stage HCC. Previous reports have indicated that AFP remains undetectable in approximately 30–35% of individuals with primary HCC, while elevated AFP levels can also be observed in individuals with normal health^25^. It is noteworthy that AFU emerged as a significantly independent factor associated with OS in early-stage HCC. Existing literature reports AFU as a specific marker for HCC, demonstrating superior sensitivity and specificity compared to AFP in the diagnosis of HCC. Particularly, AFU exhibits high accuracy in distinguishing AFP-negative cases and early-stage HCC. Consequently, the dynamic monitoring of AFU holds immense importance in the diagnosis and prognosis of early-stage HCC^26^. Besides that, ALP is also a valuable predictor of early-stage HCC patients’ DFS after curative hepatectomy in our nomogram. ALP is an important indicator of liver function and highly associated with some hepatic diseases including hepatitis, cirrhosis and HCC^27^. It has recently been reported that ALP levels could be used to monitor and predict recurrence and metastasis in HCC patients^28^. Previous studies have found that high level of ALP was related to tumor cell proliferation and epithelial-mesenchymal transition (EMT)^29,30^. Besides, the liver is highly susceptible to oxidative stress-induced damage, with ALP serving as a dependable and sensitive marker for assessing oxidative stress. The detrimental effects of oxidative stress on hepatocytes encompass lipid, protein, and DNA impairment, ultimately leading to liver injury^31^. Consequently, these processes can eventually promote the metastasis and recurrence of HCC.

Previous research has indicated a correlation between immune function and nutritional status and the prognosis of patients diagnosed with HCC^32–35^. Within our nomogram models, we have identified several influential immune and nutritional indices, namely neutrophil, monocyte, MCH, PAB, and urea, which can effectively predict prognosis. Notably, patients with low levels of neutrophils and urea, indicative of inadequate protein intake, exhibit a poorer prognosis. The tumor microenvironment is a crucial factor in the development of tumors. The immune and nutritional status, as components of the tumor microcirculation, undoubtedly impact the prognosis of patients with HCC. A growing body of evidence indicates a significant association between fundamental nutritional status, systemic inflammation, and the long-term prognosis of individuals diagnosed with cancer^36–39^. The presence of malnutrition and compromised immune function not only impacts the efficacy of treatment in individuals diagnosed with malignant tumors, but also increases the susceptibility of patients with HCC to relapse and metastasis^36^.

In recent times, metabolic disorders, specifically lipid metabolism disorders, have gained prominence as a crucial microenvironment contributing to the development of HCC^40,41^. In this study, LDL and Apo-A1, serving as indicators of hepatic lipid metabolism, emerged as significant prognostic factors for early-stage HCC. It is well-established that alterations in liver lipid metabolism are intricately linked to the onset of liver cancer, and it is plausible that non-alcoholic fatty liver disease may be recognized as a principal etiological factor for primary liver cancer in the future^42^. Furthermore, prior research has demonstrated that lipid metabolism disorders can facilitate the proliferation of tumor cells by impeding the apoptosis of hepatocellular carcinoma cells, consequently leading to an unfavorable prognosis^43^.

However, there remains scope for additional enhancements. Initially, our model predominantly relies on datasets obtained retrospectively from two Chinese institutions. Despite the satisfactory performance of the models, certain indices were not consistently gathered in certain countries, such as South Africa. The inclusion of additional cohorts from other institutions across regions may improve the predictive accuracy and universality of models. Furthermore, our models would benefit from external validation using a completely unseen and more diversity dataset, for a more accurate description of models performance. Second, though the sample size in this study is adequate, a larger sample size in conjunction with meaningful information including postoperative adjuvant treatment collected in the future may improve the accuracy of our results. Third, although, HBV infection could strongly influence on prognosis in HCC patients, the HBV-related HCC accounted for most of HCC patients in our country, which leaded it limited influence in our models. Cohort with different eitologies, multi-population and across regions will be included in the future to determine the influence of HBV infection on the prognosis in early-stage HCC patients after curative resection.

In summary, we developed and validated nomograms for predicting recurrence, especially early recurrence, and OS in patients with early-stage HCC after curative surgery. The predictive performances were superior to the common typical HCC staging systems, and they can establish patients with a high risk of recurrence or poor prognosis to provide comprehensive and accurate guidance of postoperative monitoring and adjuvant therapy after surgery, such as developing the optimal frequency of follow-up examination and individualized postoperative treatment strategies, thereby resulting in improved clinical outcomes in this group of patients.

### Supplementary Information

Supplementary Information.

## Data Availability

All data generated or analysed during this study are included in this published article.

## Abbreviations

HCC: Hepatocellular carcinoma
BCLC: Barcelona Clinic Liver Cancer
OS: Overall survival
MVI: Microvascular invasion
FHFU: The First Affiliated Hospital of Fujian Medical University
MMH: Mengchao Hepatobiliary Hospital of Fujian Medical University
CT: Computed tomography
MRI: Magnetic resonance imaging
AFP: α-Fetoprotein
DFS: Disease-free survival
C-index: Concordance index
ALP: Alkaline phosphatase
LDL: Low-density lipoprotein
Apo-A1: Apolipoprotein A1
TT: Thrombin time
MCH: Mean corpuscular haemoglobin
PAB: Prealbumin
AFU: α-Fucosidase
CI: Confidence interval
AJCC: American Joint Committee on Cancer staging system
JIS: Japan Integrated Staging Score
HKLC: Hong Kong Liver Cancer prognostic classification scheme
HBV: Hepatitis B virus
