# Supplementary material for: Novel nomograms based on microvascular invasion grade for early-stage hepatocellular carcinoma after curative hepatectomy
Source: Sci Rep. 2024 Feb 12;14:3470. doi: 10.1038/s41598-024-54260-0 (PMC10859376; doi:10.1038/s41598-024-54260-0)

Supplementary Table 1. The basic clinical characteristics of early-stage HCC patients

Clinical parameter	Total (n=703)	FHFU cohort (n=490)	MHH cohort (n=213)	p-value
Sex, male/female	580/123	404/86	175/38	0.926
Age, year	53.7±10.9	53.4±11.1	52.8±10.5	0.818
BCLC staging system, (0/A)	45/658	31/459	13/200	0.911
WBC, 10^9/L	5.2±1.6	5.0±1.9	4.9±1.5	0.942
PLT, 10^9/L	160.3±62.5	158.6±58.7	161.6±65.3	0.328
Hb, g/L	142.4±14.9	139.6±15.1	141.6±15.5	0.525
Hematocrit, %	41.5±3.9	41.3±2.7	40.4±3.3	0.803
MCV, fL	90.7±4.8	92.2±5.1	89.9±5.0	0.215
MCH, pg	31.1±2.0	31.3±1.9	31.2±1.8	0.831
Neutrophil, 10^9/L	3.1±1.2	3.0±1.1	3.1±1.2	0.887
Lymphocyte, 10^9/L	1.6±0.6	1.6±0.6	1.6±0.6	1.000
Monocyte, 10^9/L	0.4±0.1	0.4±0.1	0.4±0.1	1.000
NR, %	58.3±9.3	59.4±9.8	55.4±8.9	0.749
LR, %	31.8±8.5	32.3±8.2	32.1±8.6	0.879
MR, %	7.0±2.0	7.0±1.9	7.0±2.1	0.935
RDW, %	13.2±0.9	13.1±0.8	13.1±0.8	1.000
RBC, 10^9/L	4.6±0.6	4.6±0.6	4.6±0.5	0.684
AFP, µg/L	339.1±484.8	319±480	364.7±491.4	0.089
ALT, U/L	35.1±28.0	35.3±27	36.4±35.5	0.806
HBV infection or not	694/703	484/6	210/3	1.000
HBV DNA level, $<10^6/>10^6$ IU/mL	283/420	197/293	86/127	0.966
Albumin, g/L	42.2±3.2	42.5±3.1	42.1±3.1	1.000
Scr, µmol/L	72.1±15.6	76.9±17.1	77.4±16.5	0.727
γ-GGT, U/L	78.3±102.9	107.1±75.0	75.6±97.2	0.076
ALP, U/L	86.5±45.9	84.1±37.4	84.4±39.8	0.907
TBil, µmol/L	14.7±6.1	13.5±6.0	13.3±6.7	0.884
DBil, µmol/L	5.5±3.0	5.5±3.0	5.3±3.1	0.945
IBil, µmol/L	9.2±3.8	9.0±3.4	9.7±3.3	0.475
TBA, µmol/L	9.9±13.4	8.8±17.1	9.2±15.6	0.678
TP, g/L	69.7±5.0	74.9±4.7	75.6±4.8	0.785
ALB, g/L	42.2±3.2	42.5±3.1	42.1±3.1	0.845
GLB, g/L	27.5±4.2	29.2±5.3	27.7±4.8	0.764
ALB/GLB	1.6±0.3	1.6±0.3	1.6±0.3	1.000
PAB, mg/L	233.0±71.1	240.2±70.4	235.4±70.3	0.862
AFU, g/L	27.5±11.5	27.3±11.3	26.5±9.8	0.438
ADA, U/L	6.7±2.2	6.6±2.2	6.6±2.2	1.000
LDH, U/L	168.7±63.8	164.6±46.9	168±64.2	0.763
Urea, mmol/L	5.5±1.4	5.4±1.4	5.6±1.4	0.537
Uric acid, µmol/L	320±78.5	325±75.5	333±79.9	0.245
GLU, mmol/L	5.5±1.4	5.4±1.3	5.4±1.4	0.924

TCHO, mmol/L	4.2±0.9	4.2±0.9	4.2±0.9	1.000
TG, mmol/L	1.2±0.7	1.2±0.7	1.2±0.6	0.978
HDL, mmol/L	1.2±0.3	1.2±0.3	1.2±0.3	1.000
LDL, mmol/L	2.8±0.8	2.8±0.8	2.6±0.7	0.783
Apo-A1, g/L	119.2±30.9	117.7±29.8	121.5±31.0	0.532
Apo-B, g/L	85.6±22.0	86.7±22.5	82.3±20.5	0.375
Calcium, mmol/L	2.3±0.1	2.3±0.1	2.3±0.1	1.000
Phosphorus, mmol/L	1.1±0.2	1.1±0.2	1.1±0.2	1.000
Magnesium, mmol/L	0.9±0.1	0.9±0.1	0.9±0.1	1.000
Kalium, mmol/L	4.1±0.3	4.1±0.3	4.3±0.3	0.785
Natrium, mmol/L	141±2.4	141±2.3	141±2.4	0.942
Chlorine, mmol/L	103.0±2.9	103.1±2.7	103.0±3.1	0.921
TT, second	20.0±1.5	20.1±1.5	20.0±1.8	0.904
FIB, g/L	2.4±0.8	2.4±0.7	2.4±0.8	0.978
APTT, second	28.0±4.2	27.8±4.1	28.0±3.7	0.749
PT, second	11.7±1.1	11.9±1.0	12.3±1.2	0.532
Tumor size, centimiter	5.4±3.6	5.2±3.3	5.4±3.8	0.762
Tumor number, single/multiple	670/33	467/23	203/10	1.000
Satellite nodules, yes/no	389/314	276/214	113/100	0.422
MVI, M0/M1/M2	428/173/102	294/121/75	134/52/27	0.394
Tumor capsule, yes/no	328/375	228/262	100/113	0.919
Cirrhosis, yes/no	208/495	142/348	66/147	0.592
Follow-up time (months)	18.8±10.2	19.0±10.2	18.3±10.3	0.482
Recurrence/metastasis rates (%) (8-month/1-year/2-year/3-year)	5.8/8.1/ 11.8/12.7	6.5/9.2/ 11.7/12.5	4.2/5.6/ 9.5/10.4	0.622
Survival rate (%) (8-month/1-year/2-year/3-year)	1.7/2.4/ 3.9/4.2	1.9/2.5/ 4.1/4.4	1.8/2.2/ 3.6/4.0	1.000

FHFU, the first affiliated hospital of Fujian Medical University; MHH, Mengchao Hepatobiliary Hospital of Fujian Medical University,; BCLC staging system, Barcelona Clinic Liver Cancer staging system; WBC, white blood cell; PLT, platelet; Hb, hemoglobin; MCV, mean corpuscular volume; MCH, mean corpuscular hemoglobin; NR, neutrophil ratio; LR, lymphocyte ratio; MR, monocyte ratio; RDW, red blood cell distribution width; RBC, red blood cell; AFP, α-fetoprotein; ALT, alanine aminotransferase; HBV DNA level, hepatitis B virus deoxyribonucleic acid level; Scr, Serum creatinine; γ-GTT, γ-glutamyl transpeptidase; ALP, alkaline phophatase; TBil, total bilirubin; DBil, direct bilirubin; IBil, indirect bilirubin; TBA, total bile acid; TP, total protein; ALB, albumin; GLB, globumin; PAB, prealbumin; AFU, α-fucosidase; ADA, adenosine deaminase; LDH, lactate dehydrogenase; GLU, Glucose; TCHO, total cholesterol; TG, triglyceride; HDL, high-density lipoprotein; LDL, low-density lipoprotein; Apo-A1, apolipoprotein A1; Apo-B, apolipoprotein B; TT, thrombin time; FIB, fibrinogen; APTT, activated partial thromboplastin time; PT, prothrombin

time; MVI, microvascular invasion.

Supplementary Table 2. The C-index of the nomograms and classical staging systems

Prognostic system	Training cohort				Internal validation cohort				External validation cohort			
	DFS		OS		DFS		OS		DFS		OS	
	C-index	95%CI	C-index	95%CI	C-index	95%CI	C-index	95%CI	C-index	95%CI	C-index	95%CI
Nomograms	0.775	0.720-0.830	0.812	0.732-0.892	0.865	0.806-0.924	0.839	0.675-1.00	0.857	0.763-0.951	0.842	0.708-0.970
AJCC	0.591	0.558-0.628	0.588	0.546-0.611	0.622	0.581-0.662	0.615	0.572-0.649	0.586	0.544-0.607	0.578	0.533-0.599
BCLC	0.601	0.563-0.648	0.599	0.550-0.641	0.602	0.568-0.651	0.608	0.575-0.655	0.574	0.534-0.622	0.571	0.530-0.619
JIS	0.589	0.543-0.632	0.592	0.548-0.637	0.606	0.552-0.639	0.599	0.554-0.643	0.581	0.535-0.622	0.574	0.528-0.616
HKLC	0.595	0.562-0.629	0.612	0.568-0.632	0.625	0.581-0.649	0.619	0.577-0.638	0.558	0.528-0.580	0.541	0.512-0.568

C-index, concordance index; DFS, disease-free survival; OS, overall survival; CI, confidence interval; AJCC, American Joint Committee on Cancer; BCLC, Barcelona Clinic Liver Cancer staging system; JIS, the Japan Integrated Staging Score; HKLC, the Hong Kong Liver Cancer prognostic classification scheme.

Supplementary figure legends

Supplementary figure 1. The C-index of the nomograms and classical staging systems. A. The C-index of the DFS nomogram and classical staging systems; B. The C-index of the OS nomogram and classical staging systems. C-index, concordance index; DFS, disease-free survival; OS, overall survival; CI, confidence interval; AJCC, American Joint Committee on Cancer; BCLC, Barcelona Clinic Liver Cancer staging system; JIS, the Japan Integrated Staging Score; HKLC, the Hong Kong Liver Cancer prognostic classification scheme.

Supplementary figure 1

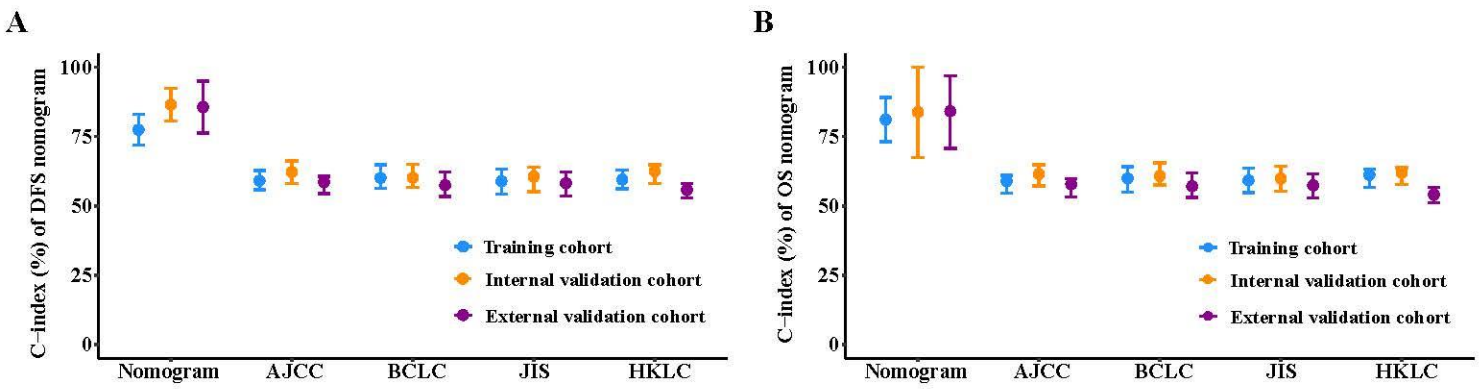

Supplement: Supplementary file 1 — Supplementary Information. [file 41598_2024_54260_MOESM1_ESM.pdf]
